# Hyperbolastic modeling of tumor growth with a combined treatment of iodoacetate and dimethylsulphoxide

**DOI:** 10.1186/1471-2407-10-509

**Published:** 2010-09-23

**Authors:** Wayne M Eby, Mohammad A Tabatabai, Zoran Bursac

**Affiliations:** 1 Department of Mathematical Sciences, Cameron University, 2800 W Gore Blvd, Lawton, OK 73505, USA; 2 Department of Biostatistics, University of Arkansas for Medical Sciences, Slot 820, Little Rock, AR 72205, USA

## Abstract

**Background:**

An understanding of growth dynamics of tumors is important in understanding progression of cancer and designing appropriate treatment strategies. We perform a comparative study of the hyperbolastic growth models with the Weibull and Gompertz models, which are prevalently used in the field of tumor growth.

**Methods:**

The hyperbolastic growth models H1, H2, and H3 are applied to growth of solid Ehrlich carcinoma under several different treatments. These are compared with results from Gompertz and Weibull models for the combined treatment.

**Results:**

The growth dynamics of the solid Ehrlich carcinoma with the combined treatment are studied using models H1, H2, and H3, and the models are highly accurate in representing the growth. The growth dynamics are also compared with the untreated tumor, the tumor treated with only iodoacetate, and the tumor treated with only dimethylsulfoxide, and the combined treatment.

**Conclusions:**

The hyperbolastic models prove to be effective in representing and analyzing the growth dynamics of the solid Ehrlich carcinoma. These models are more precise than Gompertz and Weibull and show less error for this data set. The precision of H3 allows for its use in a comparative analysis of tumor growth rates between the various treatments.

## Background

A precise mathematical formulation of biological growth is an important problem that applies to many areas of biology and can have a significant impact on understanding of growth dynamics. The application of mathematical models to understand the growth of cancer cells is a prime example, and many researchers have explored this important area. An integral part of this analysis is the choice of an appropriate growth model, and the right model can eventually aide the researcher in having a better understanding of the progression and regression of the tumor size and its associated velocity and acceleration. Sigmoidal, or logistic type growth models have been used because of the regression of the growth rate with the progression of the tumor, and the Gompertz model has been widely used in representing tumor growth. In 2005, Tabatabai *et al*. [1] introduced three flexible growth dynamic models called hyperbolastic growth models H1, H2, and H3. These models give a highly accurate estimate of parameters with low estimates of standard deviation. The hyperbolastic models have been used to analyze various biomedical problems, for instance polio data in [1], craniofacial size in [2], and dynamics of broiler growth in [3], and have always performed with a high degree of accuracy and precision. More recently these models have been shown to be the most accurate in describing dynamics of cellular proliferation for embryonic [2] stem cells. In [1] these models were also shown to be the most accurate in describing the growth of multicellular tumor spheroids in a malignant brain tumor. This paper applies the hyperbolastic models to growth of solid Ehrlich carcinoma, both in the form of growth inhibited only through the natural immune response and in the form of growth retarded through treatment with iodoacetate and dimethylsulfoxide. We are also able to apply these models in an analysis of this combined treatment.

Analysis of the growth dynamics of tumors can lead to an increased understanding in the causes for acceleration and deceleration of the rate of tumor proliferation, and furthermore an accurate quantitative knowledge of tumor growth dynamics can be applied directly to design of an optimal treatment strategy. The study of Cabrales *et al*. [4] applied the Gompertz model to describe Ehrlich tumor growth, and its effect under electrical stimulation, in order to help physicians design appropriate treatment plans. A sigmoidal model is needed in order to capture the self-limiting growth of tumors in which the growth rate decelerates with increasing age. Lala [5] stated the importance of studying the causes behind the deceleration of solid tumor growth rate, identifying possible causes to include prolonged mitotic cycle, decrease in the proliferative fraction of the tumor cells, or increases in the rate of cell loss. Recently Araujo and McElwain [6] have studied vascular collapse in relation to tumor growth rate, which has a direct effect on delivery of nutrients and delivery of anti-cancer drugs. Komarova et al. [7] have applied optimal control theory to formulate a theory in which the genetic instability and mutation within cancer cells lead to the decreased proliferation and self-limiting growth observed in solid tumors. Accurate models to describe tumor growth can lead to increased understanding of the growth dynamics and to improvements in understanding of tumor growth and improvements in treatment regimes.

The purpose of this article is to present the hyperbolastic models, and particularly H3, as highly effective and highly accurate tools in modelling the growth of solid tumors. For purposes of comparison, these models are compared with the Weibull model and particularly with the Gompertz model, which is the most prevalently used model in the field of tumor growth. Application of these growth models yields an explicit function representing the size of the tumor, as well as an explicit function representing the rate of growth. These functions allow for an analysis of the tumor growth dynamics, and we observe in the study that they are equally effective in an untreated tumor as in the cases of a single or combined treatment. Thus the study of growth dynamics can be applied to study the effectiveness of a given treatment. The article demonstrates this type of analysis in the representative case of treatment by IAA, DMSO, or both, found in Fahim *et al*. [8], and the mathematical analysis provides new insight to the combined treatment. We briefly survey the means of action of these two anti-cancer drugs.

Iodoacetate is one anti-cancer drug which may decelerate the rate of cancer cell proliferation, and it acts through disruption of tumor cell metabolism. Miko *et al*. [9] studied the impact of dactylarin as an anti-cancer agent which disables cells through inhibition of the glycolysis and energy generating pathways of the cells. The resulting impact on cancer cells through disruption of key steps in energy metabolism was observed to be similar to the effects of iodoacetate. Scatena *et al*. [10] discuss the importance of drugs which disturb cancer cell glycolysis in cancer therapies. They describe five potential actions of such drugs on cancer cells and propose further study of the unique metabolism displayed by cancer cells. Boros et al. [11] discuss the role of pentose phosphate pathways (PPP) in tumor proliferation and propose to investigate newer cancer treatments blocking specific reactions within the PPP. Badwey and Karnovshy [12] report that iodoacetate inhibits two important enzymes in the oxidative stage with the PPP. As an anti-cancer drug iodoacetate acts through disruption of metabolism within the tumor cells to prevent proliferation.

The use of dimethylsulphoxide to slow the proliferation of cancer is based on a different method of action, primarily the stimulation of the quiescent phase within the cancer cells. Dimethylsulfoxide is commonly used as a solvent because of its ease in passing cellular and vascular membranes, and it is well known to induce differentiation. Higgins and O'Donnell [13] noticed a dose-dependent reduction in proliferation of murine hepatoma cells exposed to dimethylsulfoxide. Higgins [14] determined that the dimethylsulfoxide suppresses cellular proliferation through a substantial reduction in the cellular RNA content and stimulation of the quiescence in the exposed cells.

Fahim *et al*. [8] propose to use both iodoacetate and dimethylsulfoxide in a combined treatment in order to combine the effects of each drug. The induction of cellular quiescence by the DMSO and the disruption of cellular metabolism by IAA affect two different aspects of cellular growth, and they are expected to combine for a greater effect.

## Methods

Tabatabai *et al*. [1] introduced the hyperbolastic growth models. The first of the three differential equations is called the hyperbolastic growth rate of type I (H1) which has the equation of the form

(1) $$\frac{dP(t)}{dt}=\frac{P(t)}{M}(M-P(t))\left(\delta +\frac{\theta }{\sqrt{1+t^{2}}}\right)$$

With the initial condition

$$P(t_{0})=P_{0}$$

where *P*(*t*) is the population size at time *t*, the constant *M *is the parameter representing carrying capacity, and *δ *and *θ *jointly determine the growth rate. The magnitude of the parameter θ represents the distance from a symmetric sigmoidal distribution. Solving the equation (1) for the population size *P *gives

(2) $$P(t)=\frac{M}{1+\alpha EXP\left[-\delta t-\theta arcsinh(t)\right]}$$

where

$$\alpha =\frac{M-P_{0}}{P_{0}}EXP\left[\delta t_{0}+\theta arcsinh\left(t_{0}\right)\right]$$

and arcsinh(t) is the inverse hyperbolic sine function of t. We call the function *P*(*t*) of equation (2) the hyperbolastic growth model of type I or simply H1.

The second differential equation which was developed earlier is called the hyperbolastic growth rate of type II (H2) and has the form

(3) $$\frac{dP(t)}{dt}=\alpha \delta \gamma P^{2}(t)t^{\gamma -1}tanh\left[\frac{M-P(t)}{\alpha P(t)}\right]/M$$

with the initial condition *P*(*t*_0_) = *P*_0 _and *γ *> 0, where tanh[.] stands for hyperbolic tangent function. *M *is the parameter representing the carrying capacity, and the parameters *δ *and *γ *jointly determine the growth rate. The parameter γ represents acceleration in the time course. Solving the equation (3) for population size *P *gives

(4) $$P(t)=\frac{M}{1+\alpha arcsinh\left[EXP\left(-\delta t^{\gamma }\right)\right]}$$

where

$$\alpha =\frac{M-P_{0}}{P_{0}arcsinh\left[EXP\left(-\delta t_{0}^{\gamma }\right)\right]}$$

Finally, we consider the third growth curve through the following nonlinear hyperbolastic differential equation of the form

(5) $$\frac{dP(t)}{dt}=(M-P(t))\left(\delta \gamma t^{\gamma -1}+\frac{\theta }{\sqrt{1+\theta^{2}t^{2}}}\right)$$

with the initial condition *P*(*t*_0_) = *P*_0_. The parameter M represents the carrying capacity, and the parameters *δ*, *γ*, and *θ *jointly determine the growth rate. The parameter γ represents acceleration of the time scale, while the size of θ represents distance from a symmetric sigmoidal curve. We refer to the model (5) as the hyperbolastic ordinary differential equation of type III or H3. The solution to the equation (5) is

(6) $$P(t)=M-\alpha EXP\left[-\delta t^{\gamma }-arcsinh(\theta t)\right]$$

where

$$\alpha =(M-P_{0})EXP\left[\delta t_{0}^{\gamma }+arcsinh(\theta t_{0})\right]$$

We call the function *P*(*t*) of equation (6) the hyperbolastic growth model of type III or simply H3. If necessary, one can introduce shift or delay parameters in any or all hyperbolastic growth models.

The parameters are estimated using computational software SPSS and Mathematica to produce a best fit to the experimental data. It is also possible to use the SAS package. For instance the method of non-linear least squares regression for the H3 model (6) is used to determine the model parameters. Using SPSS, the input data can be analyzed using the Nonlinear Regression module, found under Analyze and Regression. After entering formula (6) into the box for Model Expression, it is then necessary to enter initial value estimates for the parameters. In SPSS, the arcsinh(x) function must be entered using its definition in terms of logarithms: $arcsinh(x)=\ln(x+\sqrt{1+x^{2}})$. An example of the source code used to estimate the parameters in SAS can be found in the additional file of [1].

Note that *P*'(*t*) and *P*"(*t*), the velocity and acceleration of growth, can be explicitly determined, as functions of time, once the parameters for *P*(*t*) have been determined. Mathematica is an effective tool for computation of *P*'(*t*) and *P*"(*t*), as well as for their use in studying cancer growth dynamics. Description of rate of growth as an explicit function *P*'(*t*) is more accurate and realistic than use of a static parameter, for instance. The explicit functions and *P*"(*t*) allow for a deeper analysis of the growth dynamics.

The same methods of analysis and curve fitting will also be applied to the data using the Gompertz model, of the following form:

P(t)=αEXP[bEXP[ct]]

where

$$\alpha =P_{0}*EXP[-bEXP[ct_{0}]]$$.

for initial conditions *P*(*t*_0_) = *P*_0 _The parameters b and c satisfy b, c < 0. These methods will also be applied to the Weibull model of the form

$$P(t)=M-\alpha EXP(-\beta t^{\gamma })$$

where M, β, and γ are model parameters and

$$\alpha =(M-P_{0})EXP(\beta t_{0}{}^{\gamma }).$$

## Results

Fahim et al. [8] analyzed the survival times of mice by investigating the effect of the anti- tumor treatments iodoacetate, dimethylsulfoxide, and the combined treatment effect of both iodoacetate and dimethylsulfoxide on the retardation of solid Ehrlich carcinoma. As mentioned in the background, they demonstrated that the disruption of cell metabolism by iodoacetate and the stimulation of cellular senescence by dimethylsulfoxide complement one another and produce a greater combined effect. The researchers recommend the combined treatment by iodoacetate (IAA) and dimethylsulphxide (DMSO) as an appropriate action to be taken, and their data supports the strengthened effect of combining these two anti-cancer chemicals.

In this section, we first apply the hyperbolastic growth models H1, H2, and H3 to their data in order to obtain predictive growth functions for the growth dynamics of the mean tumor weights after receiving the combined treatment of iodoacetate and dimethylsulfoxide. For purposes of comparison, the Gompertz and Weibull models are also fit to the same data. Table 1 gives the parameter values, as estimated using SPSS, for all the models, hyperbolastic, Gompertz, and Weibull. In addition Table 2 compares the observed value with the predicted value for each of the models H1, H2, H3, Gompertz, and Weibull.

**Table 1:** Parameter estimates for the Solid Ehrlich Carcinoma treated with combined IAA and DMSO using models H1, H2, H3, Gompertz, and Weibull.

Model	Parameter	Estimate	**Std. Dev**.	95% Confidence Interval	
				
				Lower Bound	Upper Bound
	**M**	8.298	0.190	7.893	8.703
**H1**	**δ**	0.087	0.006	0.074	0.100
	**θ**	-0.206	0.141	-0.506	0.094

	**M**	8.223	0.204	7.787	8.659
**H2**	**δ**	0.055	0.014	0.025	0.085
	**γ**	1.088	0.061	0.958	1.129

	**M**	7.533	0.098	7.322	7.744
**H3**	**δ**	3.594E-9	0.000	-4.822E-9	1.201E-8
	**γ**	4.712	0.265	4.143	5.281
	**θ**	0.004	0.001	0.003	0.005

**Gompertz**	**b**	-5.418	0.071	-5.570	-5.267
	**c**	-0.025	0.002	-0.028	-0.022

	**M**	8.024	0.235	7.523	8.524
**Weibull**	**β**	-8.579E-7	0.000	-1.807E-6	9.076E-8
	**γ**	3.399	0.139	3.103	3.696

**Table 2:** Observed and estimated values for the weight of Solid Ehrlich Carcinoma treated with combined IAA and DMSO.

Time	Observed weight	H3 Estimated weight	H1 Estimated weight	H2 Estimated weight	Weibull Estimated weight	Gompertz Estimated weight
9.00	0.21	0.21	0.21	0.21	0.21	0.21
13.0	0.39	0.33	0.27	0.27	0.24	0.32
17.0	0.49	0.46	0.36	0.36	0.30	0.46
21.0	0.65	0.59	0.48	0.49	0.40	0.64
29.0	0.89	0.96	0.86	0.87	0.80	1.15
33.0	1.13	1.21	1.15	1.15	1.11	1.48
37.0	1.49	1.53	1.51	1.51	1.51	1.85
40.0	1.89	1.83	1.83	1.84	1.86	2.17
43.0	2.31	2.18	2.21	2.22	2.26	2.50
49.0	3.05	3.04	3.01	3.12	3.17	3.24
55.0	4.05	4.08	4.10	4.15	4.16	4.04
57.0	4.51	4.45	4.45	4.50	4.50	4.32
60.0	4.96	5.00	4.96	5.01	5.00	4.74
66.0	5.91	6.01	5.90	5.95	5.92	5.61
72.0	6.86	6.78	6.66	6.71	6.68	6.49
74.0	7.09	6.97	6.87	6.91	6.90	6.78
78.0	7.21	7.24	7.23	7.26	7.25	7.35
82.0	7.35	7.40	7.50	7.52	7.52	7.91

**Figure 1:**
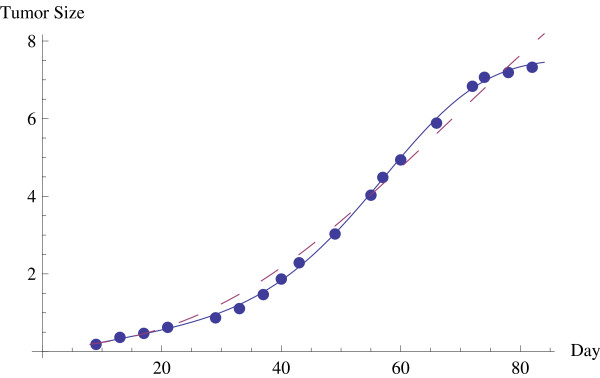
**Growth of tumor biomass under combined IAA/DMSO treatment compared to H3 and Gompertz growth curve estimates**.

All three of the hyperbolastic models are seen to be highly accurate in representing the growth of the treated tumors, while the model H3 shows a higher accuracy than the others. These all compare favourably to Gompertz, as can be seen in Table 2 above. See also Figure 1 below, which shows the actual data and a comparison of the fit between H3 and Gompertz growth curves. Table 3 below gives a comparison of accuracies in the estimates given by hyperbolastic models H1 to H3 and also the Weibull and Gompertz models. Note that although these results are for the data set for solid Ehrlich carcinoma with a combined treatment of IAA and DMSO, the results for each of the treated cases, IAA alone and DMSO alone, and for the untreated case are similar. The measures of accuracy in Table 3 are Akaike information criterion (AIC), residual mean square (RMS), R^2^, and mean absolute relative error (MARE). The measurement of AIC is used in model selection to compare different models with different number of parameters. The model with the lowest AIC value is considered the best.

**Table 3:** Accuracy of models in estimating growth of solid Ehrlich carcionma with combined treatment of IAA and DMSO.

	H3	H2	H1	Weibull	Gompertz
**AIC**	-37.23951	-25.47660	-24.96665	-18.38632	7.53500

**RMS**	0.006	0.012	0.012	0.017	0.076

**R**^**2**^	0.999	0.999	0.999	0.998	0.990

**MARE**	0.0367	0.0598	0.0594	0.0819	0.0959

**Figure 2:**
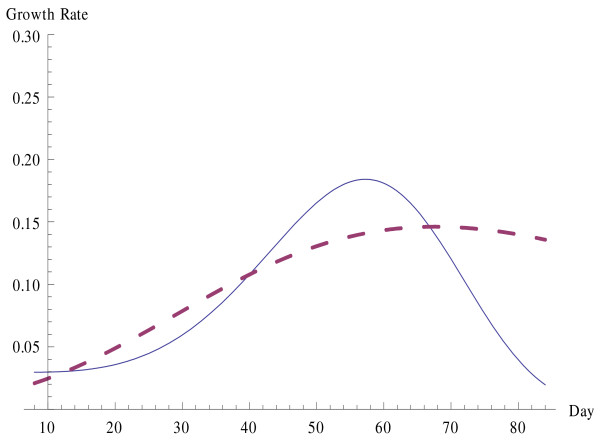
**Rate of growth of tumor biomass under combined IAA/DMSO treatment as measured by H3 and Gompertz growth curves**.

Clearly the best of the above models is H3, by all of the measures of accuracy given in Table 3. This model will be compared further with the Gompertz model, which is the model most prevalently used in tumor growth modelling. Observe in Tables 2 and 3 and Figures 1 and 2 that for the current data set the estimated values given by Gompertz are really some distance from the actual values. Figure 1 compares the actual values to the H3 and Gompertz estimates. The H3 growth curve is solid while the Gompertz curve is dashed. This inaccuracy is further magnified in measurement of the growth rate, whereas an accurate estimate of this rate is critical in applications and in design of an optimal treatment regimen. Figure 2 shows a comparison of the growth rates with H3 a solid curve and Gompertz a dashed curve.

The growth rate for the tumor weight of the Solid Ehrlich Carcinoma for a combined treatment group using hyperbolastic H1 reaches its maximum of 0.172765 grams per day on the day 55.4873 but for the hyperbolastic H2 and H3, the maximum rates are 0.175028 and 0.184104 grams per day and occur on the days 55.2494 and 57.2509 respectively. All of these are comparable in both the size of the maximum and the time of occurrence. Although all of these give results that are fairly similar and close to the exact values, the estimate by Gompertz is considerably off the mark, estimating a maximum rate of 0.146143 grams per day on day 67.5891. The distance of Gompertz from the correct prediction of the rate of growth can be clearly seen in Figure 2 above.

**Figure 3:**
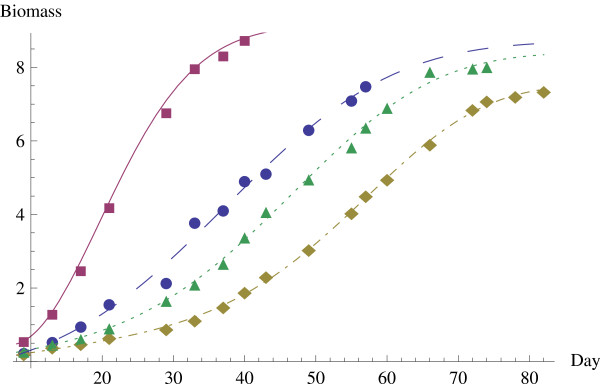
**Growth of tumor biomass for each treatment using H3 model**.

**Figure 4:**
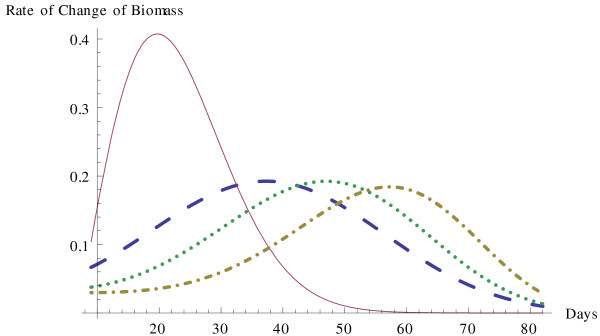
**Rate of growth for tumor biomass for each treatment using H3 model**.

Using the most accurate of the above models, H3, we also make a comparative analysis of the growth of the solid Ehrlich carcinoma cells under the various treatments: combined treatment of iodoacetate and dimethylsulfoxide, treatment by only iodoacetate, treatment by only dimethylsulfoxide, and the control of no treatment. The time course of tumor growth of each of these is shown in Figure 3. The solid curve represents the untreated tumors, the dashed curve represents treatment by IAA, the dotted curve represents treatment by DMSO, and the dotted and dashed curve represents the combined treatment. The observed values are plotted together with these growth curves in Figure 3, and the graph clearly indicates the H3 model estimates the data accurately. In these other treatments the tumor growth stopped sooner than in the combined treatment, at the time of death of the experimental mice. In all cases, the rate of growth initially increases with increasing tumor size. The deceleration of this rate with increasing age and size of the tumor is the object of study proposed by Lala [5]. Our analysis gives a mathematical representation of the dynamics of this growth rate. Figure 4 shows the rates of growth for each of these cases. The solid curve represents the untreated tumors, the dashed curve represents treatment by IAA, the dotted curve represents treatment by DMSO, and the dotted and dashed curve represents the combined treatment. The untreated tumors clearly reach a much higher rate of increase, more than two times as large, also occurring much earlier. In all of the treated cases, the maximum rate of increase is approximately the same. However there is a clear difference in when the maximum occurs, with the more effective treatments keeping the rate lower for longer periods of time and having a maximum occurring later in time. In the combined treatment of IAA and DMSO, the delays in achieving the maximum rate of growth are also combined, leading to a longer period with a slow rate of growth.

In the untreated tumors, the maximum rate of growth is 0.407303 grams per day, occurring on day 19.6933. For the tumors treated with only iodoacetate, the maximum rate of growth is 0.192412 grams per day, occurring on day 37.3535. The tumors treated with only dimethylsulfoxide have a maximum rate of growth of 0.192293 grams per day, occurring on day 46.9909. Finally the tumors with the combined treatment of iodoacetate and dimethylsulfoxide have a maximum rate of growth of 0.184104 grams per day, occurring on day 57.2509. It is interesting that all of the treated cases display approximately the same level for the maximum rate of growth. In all cases, this rate is less than half of the untreated tumors, displaying the effectiveness of all three treatments. However, the treatment with iodoacetate is the least effective of these, with the maximum occurring sooner, at time 37.3535 days, while the combined treatment is the most effective, as the rate remains lower for a longer period of time and does not reach the maximum until day 57.2509. The article of Fahim *et al*. [8] claimed a potential synergism between the iodoacetate and dimethylsulfoxide in the combined treatment. The combining of the effects of these two treatments can be seen in the graphs. The maximum rate in the combined treatment only lowers slightly, however the time of that maximum is shifted further to the right in time, as if the two treatments are combining their effects.

## Discussion

In the comparison of models we observed that the hyperbolastic models perform with a greater degree of accuracy than either the Weibull or Gompertz models. In particular H3 has the highest level of accuracy, considerably better than any of the other competing models. The hyperbolastic models were designed for flexibility in modelling biological growth, allowing for flexibility in the time at which the growth becomes self-limiting and flexibility in the manner in which the rate goes to zero as the population approaches the carrying capacity. Growth of multicellular tumor spheroids is well known to be self-limiting and fits well into this form of growth. The H3 model is particularly effective, as has also been the case in other comparative studies, i.e. [1], [2], [3], and its strength is in its flexibility to fit biological growth curves. Expressing H3 in the form

$$P(t)=M-\frac{\alpha }{\theta t+\sqrt{1+{\left(\theta t\right)}^{2}}}EXP[-\delta t^{\gamma }]$$

we see that some of the flexibility originates from the time dependence in the term

$$\frac{\alpha }{\theta t+\sqrt{1+{\left(\theta t\right)}^{2}}}$$

which allows the population to approach M more flexibly than using only a static α, but also reduces to a static α when θ = 0.

In assessing a treatment it is important to use the most accurate model and find the most accurate predictions. In comparing the most accurate model H3 with Gompertz, the model most prevalently used, we observed that H3 performs significantly better, while the results according to the Gompertz model can be off by a significant amount. Particularly in comparison of the rates of growth, as in Figure 4, the distance between the Gompertz model and the actual values is considerable. With the accuracy of the H3 model, we can be certain that the comparative analysis of the treatments, as illustrated in Figure 3 and Figure 4, is representative of the actual data, although the same would not be true for the Gompertz model. A comparative analysis of treatment of solid Ehrlich carcinoma by treatments of IAA alone, DMSO alone, or a combined treatment of IAA and DMSO revealed that each of these treatments significantly delayed tumor growth, cutting the maximum rate of growth to less than half the original untreated tumor, and delaying the time for the maximum rate of growth from 19.6933 days to anywhere from 37.3535 to 57.2509, i.e. by a factor of two to three. This yields a good quantitative assessment of the treatment effectiveness. Furthermore the analysis helps to describe the means by which the two treatments combine. In the combined treatment, the maximum rate of growth is depressed only slightly, but the main impact comes in the delay of the time of the maximum rate, to approximately 10 to 20 days later than the single treatments.

An accurate representation and understanding of growth dynamics of tumors can be used by physicians in designing optimal treatment strategies or by scientists in analyzing the factors leading to deceleration of growth in multicellular tumor spheroids. In order to have the most accurate information and make the best decisions, it is valuable to have the increased precision available in the H3 model. In designing a treatment plan it is helpful to know the magnitude of growth and the time at which the growth rate is maximized, and H3 can estimate these more accurately than other models. It is also possible to measure the effect of any given treatment on the tumor growth, as illustrated in Figures 3 and 4 and the associated discussion. Furthermore a multivariable version of the H3 model is available, and the multivariable model can measure the effects of explanatory variables, such as the level of a given drug applied in a treatment.

## Conclusions

All three models H1, H2, and H3 proved successful in modeling solid Ehrlich carcinoma growth under the combined treatment with iodoacetate and dimethylsulfoxide, while the hyperbolastic model H3 had the least Absolute Relative Error as well as the least Residual Mean Squared Error. The R-Squared for all hyperbolastic models was 0.999. The Mean Absolute Relative Error was also very small, ranging from 0.0367 for H3 to 0.0598 for H2. The most accurate model, H3, was used to compare growth under various treatments, for the cases of the combined treatment, treatment with iodoacetate alone, treatment with dimethylsulfoxide alone, and the untreated control. The comparative analysis of the growth rates shows comparable maximum rates of growth of the tumors for all three cases of treated tumors, less than half of the maximum growth rate for the untreated tumor. But in the more effective treatments, the tumor grows more slowly at the outset, and the maximum rate is delayed to a later time. In the combined treatment of IAA and DMSO, the delays in achieving the maximum growth rates are combined to give an even longer period of slow growth before the maximum growth rate is achieved. This analysis supports the claims of Fahim *et al*. [8] that the anti-cancer drugs iodoacetate and dimethylsulfoxide can combine to form a greater effect because their methods of action are complementary. The hyperbolastic models prove to be effective in representing and analyzing the tumor cell growth dynamics.

## Abbreviations used

IAA: Iodoacetate; DMSO: dimethylsulfoxide; PPP: pentose phosphate pathway; H1: hyperbolastic growth model of type I; H2: hyperbolastic growth model of type II; H3: hyperbolastic growth model of type III

## Competing interests

The authors declare that they have no competing interests.

## Authors' contributions

WME made the comparative analysis of the treatments and wrote the paper, read and approved final version. MT designed the model and made the analysis of the combined treatment data in models H1, H2, H3, Weibull, and Gompertz, read and approved final version. ZB assisted with the analysis and consulted on the results, read and approved final version.

## Pre-publication history

The pre-publication history for this paper can be accessed here:

http://www.biomedcentral.com/1471-2407/10/509/prepub
